# Evolutionarily conserved odorant receptor function questions ecological context of octenol role in mosquitoes

**DOI:** 10.1038/srep37330

**Published:** 2016-11-16

**Authors:** Amir Dekel, Ronald J. Pitts, Esther Yakir, Jonathan D. Bohbot

**Affiliations:** 1 Department of Entomology, The Hebrew University of Jerusalem, Rehovot 76100, Israel; 2 Department of Biological Sciences, Vanderbilt University, Nashville, Tennessee, USA

## Abstract

Olfaction is a key insect adaptation to a wide range of habitats. In the last thirty years, the detection of octenol by blood-feeding insects has been primarily understood in the context of animal host-seeking. The recent discovery of a conserved octenol receptor gene in the strictly nectar-feeding elephant mosquito *Toxorhynchites amboinensis (TaOr8*) suggests a different biological role. Here, we show that TaOR8 is a functional ortholog of its counterparts in blood-feeding mosquitoes displaying selectivity towards the (*R*)-enantiomer of octenol and susceptibility to the insect repellent DEET. These findings suggest that while the function of OR8 has been maintained throughout mosquito evolution, the context in which this receptor is operating has diverged in blood and nectar-feeding mosquitoes.

The molecular mechanisms by which insects detect odor cues involve many gene families of which odorant receptors (*Ors*) play a major role^1^. The interactions between ORs and their ligands (biochemical function) and the ecological contexts in which these interactions take place are fascinating relationships to be explored. With the exception of pheromone receptors, whose roles are well understood, there is little knowledge of how insects detect and process specific olfactory cues that are important to their life histories.

The survival of most mosquitoes depends on locating animal hosts, resting and oviposition sites, as well as suitable sources of nectar (Fig. 1a)^2^. The role of (*R*)-(−)-1-octen-3-ol (thereafter termed (*R*)-octenol) is of particular interest for several reasons. First identified as a tsetse attractant released by cattle^3^, octenol has also been shown to be present in human sweat^4^ and to attract mosquitoes^5^. The identification of a labeled pathway for this compound in all blood feeding mosquitoes^6^,^7^,^8^ suggests a role in animal host seeking as well^2^. However, the behavioral significance of octenol is complex, species specific^5^,^9^,^10^ and generally poorly understood^11^. Octenol may require the concomitant presence of other cues such as CO_2_^5^. Octenol seems to be an attractant for *Anopheles* and *Aedes* but in *Culex*, octenol elicits little to no attractive effects^12^ or repels this mosquito in a host-feeding context^13^.

Octenol may play a role in nectar-seeking as proposed by earlier authors^14^. For instance, leaves and flowers of the wild sage *Lantana camara* release (*R*)-octenol^15^ and are known to attract mosquitoes^16^. Other observations suggest that octenol is involved in behaviors other than animal host-seeking. Non-blood-feeding male *Ae. aegypti* exhibit octenol-sensitive basconic sensilla^17^ and express the *Or8* gene (*AaOr8*)^18^. The role of OR8 in *Culex quinquefasciatus* mosquitoes is unclear as multiple OR8 paralogs are activated by high doses of octenol^13^,^19^. This mosquito also shows a marked preference for birds^20^, which are not known to release octenol^21^. However, these observations may be consistent with animal host-seeking if one considers that male *Ae. aegypti* have been found in proximity to hosts, perhaps as a means to increase their likelihood to locate a mate, and that octenol has not been excluded as a bird emanation. In any case, the 145–200 million years old conservation of the octenol receptor OR8, in Culicinae and Anophelinae mosquitoes^18^,^22^ underscores its importance perhaps in multiple ecological contexts.

The recent discovery of the OR repertoire in the Elephant mosquitoes *Toxorhynchites* might be valuable to explore the role of octenol since animal host-seeking is not part of their behavior^23^,^24^ (Fig. 1a). The *Toxorhynchites* group, which belongs to the Culicinae subfamily, separated from the *Aedes* and *Culex* lineages about 38–54 million years ago^22^,^25^. *T. amboinensis* belong to a small group of nectar-feeding mosquitoes^26^, which share a majority of *Or* homologs with *Ae. aegypti* including *Or8 (TaOr8*)^27^. Additionally, *T. amboinensis* and *Ae. aegypti* express *Or8* at similar levels in the maxillary palps suggesting a conserved key role in the life cycle of adult mosquitoes. Resolving the tuning properties of TaOR8 may clarify the role of this receptor outside animal host-seeking paradigms. Using a cell-based functional assay, we show that TaOR8’s response to octenol is highly sensitive, enantioselective, inhibited by the insect repellent DEET and odorant specific. Common ancestral origin and functional conservation support TaOR8 as a functional ortholog of the *Ae. Aegypti*, *Anopheles gambiae* and *Culex quinquefasciatus* OR8s. These features provide evidence that TaOR8 is an octenol receptor whose ecological role is unknown but excludes animal host-seeking. These findings question the ecological role traditionally ascribed to the OR8/(*R*)-octenol partner in blood-feeding mosquitoes and suggest that octenol may be useful to mosquitoes in multiple contexts beyond animal host-seeking.

## Results

### High amino-acid sequence conservation of OR8 in blood and nectar feeding mosquitoes

**Figure 1:**
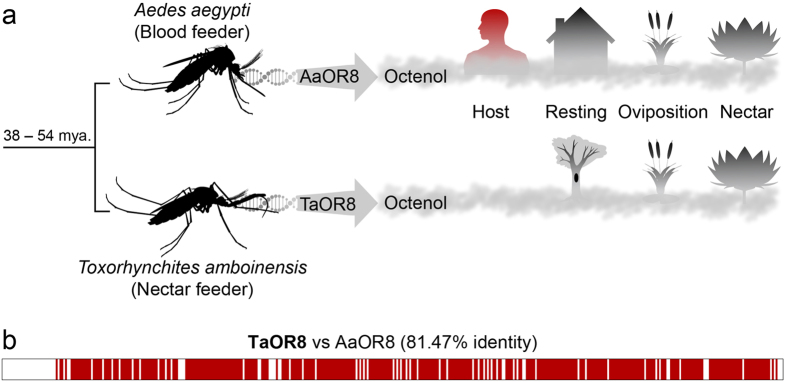
Ecological context of OR8 function. **(a)** *Aedes aegypti* and *Toxorhynchites* diverged 40 million years (MY) ago. Both insects may use octenol in overlapping and different contexts. **(b)** TaOR8 and AaOR8 share 81.47% amino-acid sequence identity (red).

Despite 38–54 million years of evolution since the *Aedes*-*Toxorhynchites* split and different ecological requirements (Fig. 1a), TaOR8 and AaOR8 exhibit high peptide sequence conservation (Fig. 1b). TaOr8 encodes a 394 amino-acid protein (1185 nucleotides including the stop codon) sharing 81% overall amino-acid identity with AaOR8^28^ (Fig. 1b). Previous functional analysis of AaOR8 was carried out with a gene (1269 nucleotides including stop codon) encoding a 422 amino-acid protein. While amino-acid divergence is evenly distributed throughout the peptide sequence, highest amino-acid diversity is highest on the N-terminus of AaOR8, which exhibits an extra 26 amino-acids.

### TaOR8 is enantioselective

**Figure 2:**
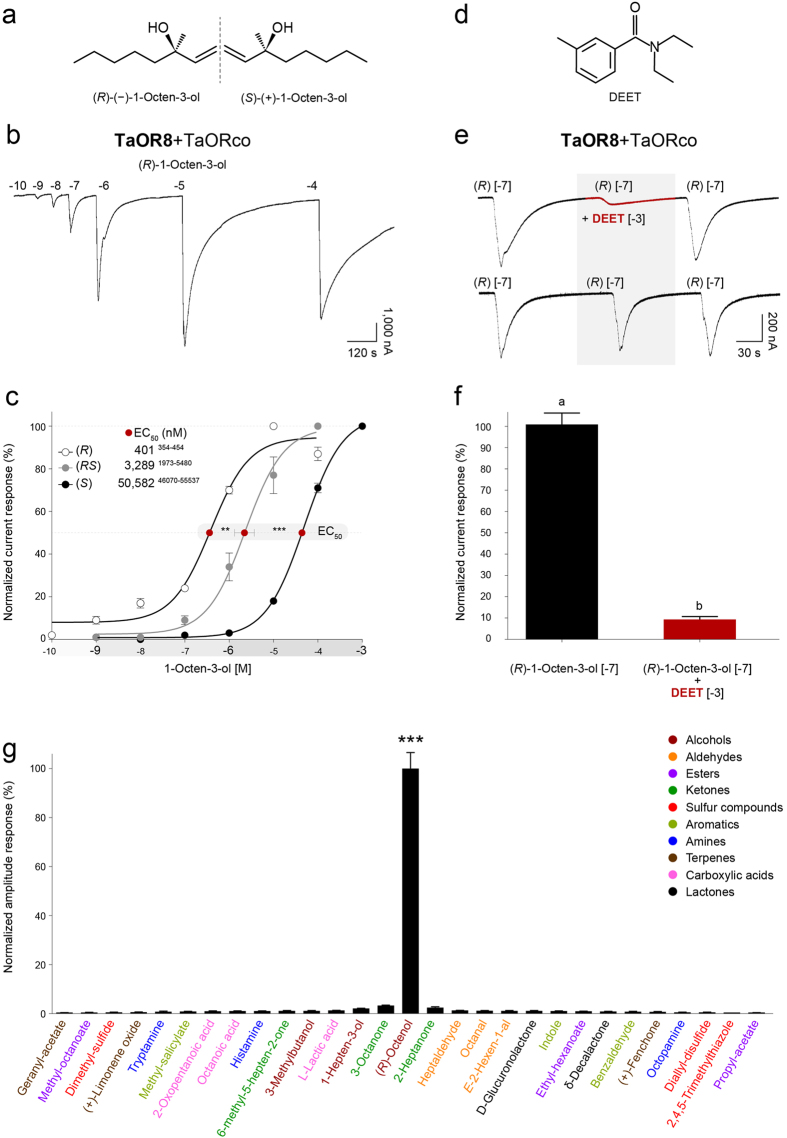
Functional analysis of TaOR8. **(a)** 1-Octen-3-ol occurs in two enantiomeric forms, (*R*)-(−)-1-octen-3-ol and (*S*)-(+)-1-octen-3-ol. **(b)** Representative current trace elicited by increasing concentrations of (*R*)-(−)-1-octen-3-ol recorded from *Xenopus* oocytes co-expressing the *TaOr8* and *TaORco* receptor complex. **(c)** Concentration-response relationships of TaOR8+TaORco elicited by (*R*)-(−)-1-octen-3-ol [(*R*), open circle, n = 5], (*S*)-(+)-1-octen-3-ol [(*S*), grey circles, n = 5) and (*RS*)-1-octen-3-ol [(RS), black circles, n = 6]. Responses were normalized to the maximum response. Extrapolated EC_50_ values are shown with red circles. Lower and upper EC50 values (standard error) are in upper case. Asterisks represent statistically significant differences of the OR responses (one-way ANOVA followed by Tukey’s post test; **P < 0.01 and ***P < 0.001). Odorant concentrations were plotted on a logarithmic scale. Each point represents the mean and error bars indicate s.e.m. **(d)** N,N-Diethyl-meta-toluamide, commonly called DEET, is a synthetic insect repellent. (**e**) DEET inhibits the response of OR8 to octenol: Representative current traces of oocytes expressing TaOR8+TaORco elicited by 10^−7^ M (*R*)-(−)-1-octen-3-ol alone or in combination with 10^−3^ M DEET. **(f)** Normalized responses of TaOR8+TaORco to 10^−7^ M (*R*)-(−)-1-octen-3-ol alone or in combination with 10^−3^ M DEET. DEET’s effect was statistically significant (Student’s t-test, P < 0.01, n = 5–7). (**g**) (*R*)-(−)-1-octen-3-ol is a potent TaOR8 activator (one-way ANOVA followed by Tukey’s post test; ***P < 0.0001). Mean responses (±s.e.m., n = 6) to 400 nM of 28 odorants were normalized to (*R*)-octenol.

Octenol is a chiral compound composed of the (*R*)-(−)-1-octen-3-ol and (*S*)-(+)-1-octen-3-ol enantiomers (Fig. 2a). The (*R*) enantiomer is the predominant form^15^,^29^,^30^ found in nature. In order to investigate whether TaOR8 is a functional ortholog of AaOR8, we expressed TaOR8 in combination with TaORco in *Xenopus laevis* oocytes and recorded the responses of this receptor complex to the (*R*), (*S*) and racemic mixture (*RS*) of 1-octen-3-ol using the two-microelectrode voltage clamp technique. An example of a current trace is shown in Fig. 2b. The resulting electrophysiological responses were fitted to sigmoid curves (Fig. 2c). Extrapolated EC_50_ values show that the (*R*) enantiomer (EC_50_: 401 nM) is approximately 8 and 126 times more potent than the (*RS*) mixture (EC_50_: 3,289 nM) and the (*S*) enantiomer (EC_50_: 50,582 nM), respectively. Although the (*S*) contained <0.1% or no (*R*) at all, we cannot exclude the possibility that TaOR8 response was elicited by trace amount of the (*R*) enantiomer. Sensitivity in the nanomolar range for (*R*)-octenol supports a cognate receptor ligand relationship, which is comparable to pheromone receptor-pheromone pairs^31^.

### DEET inhibits TaOR8’s response to (R)-octenol

To further confirm that both mosquito receptors are functional orthologs, we tested the inhibitory effect of DEET (10^−3^ M) (Fig. 2a) on the TaOR8 response to a non-saturating concentration of (*R*)-octenol (10^−7^ M), as carried out previously with AaOR8^32^. Despite the alleged masking effect of DEET on octenol shown in single cell recordings from olfactory receptor neurons^33^, we have previously shown that no such effects occur in solution^34^. An example of a current trace is shown in Fig. 2e. DEET reduced TaOR8 activation by 90% (Fig. 2f). Following DEET exposure, TaOR8 response to octenol returned to baseline. Response to (*R*)-octenol alone did not differ before and after exposure to the (*R*)-octenol-DEET mix, indicating no adaptive effect (Fig. 2f). Applying three consecutive doses of 10^−7^ M (*R*)-octenol elicited identical TaOR8 responses, excluding a potential position effect.

### TaOR8 is narrowly tuned to (R)-octenol

We used a panel of 29 compounds including (*R*)-octenol) belonging to 10 classes of organic compounds (alcohols, aldehydes, esters, ketones, sulfur compounds, aromatics, amines, terpenes, carboxylic acids and lactones) to explore the odor space of TaOR8 (Fig. 2g). All compounds were delivered for 8 s at a concentration of 400 nM, which corresponds to the EC_50_ value of (*R*)-octenol. (*R*)-octenol was the most potent ligand eliciting a response 30 times higher than the next most potent chemical 3-octanone, also an 8-carbon aliphatic compound, and 46 times higher than 1-hepten-3-ol, which is identical to octenol except for one carbon shorter.

## Discussion

Our major objective was to functionally characterize OR8 from a non-blood feeding mosquito as a means to explore its potential biological role in blood-feeding mosquitoes. This interest was motivated by the recent discovery of a conserved octenol receptor in *T. amboinensis* solely expressed in the maxillary palp^27^, which suggested that this receptor might be a functional octenol receptor operating in a context other than animal host-seeking.

This study shows that TaOR8 and AaOR8 are functional orthologs as both (i) share a high level of sequence identity, (ii) are expressed in the maxillary palps, (iii) exhibit high sensitivity (nanomolar range) towards (*R*)-octenol, (iv) feature a susceptibility to DEET inhibition, (v) are narrowly tuned to (*R*)-octenol. Such evolutionarily conservation of receptor biochemical function in *Toxorhynchites* is surprising since this species does not animal host-seek^23^,^24^. Indeed, considering that 95% of mosquito species are blood-feeders and assuming that octenol plays a role in animal host-seeking, expecting a tuning shift in TaOR8 would have been a reasonable expectation. Maintenance of the octenol-receptor phenotype in a non-animal host-seeking mosquito supports a role in locating resting/oviposition sites, nectar sources or other contexts (Fig. 1a). Perhaps more intriguing is the possibility that OR8 in blood-feeding mosquitoes may also play a role outside animal host-seeking^14^. These findings suggest that conservation of biochemical function does not necessarily translate into conserved behaviors, which underscores the role of the brain in determining their ecological contexts.

Our results support a role of TaOR8 outside an animal host-seeking context. Since TaOR8 requires ORco, this is consistent with the discovery that *orco* is not only involved with animal host selection but also with the detection of honey, which contains nectar metabolites (DeGennaro *et al.*, 2013). Identifying other contexts in which this compound is used by mosquitoes will be challenging, as octenol is a common environmental volatile that may serve multiple roles in mosquito behavior. Octenol is synthesized by fungi^35^,^36^, plants^15^,^37^,^38^,^39^ and is also released by vertebrates^3^,^40^,^41^. However, whether animals possess a biosynthetic pathway to produce this chemical is unknown and it is possible that its occurrence in animal secretions results from microbial activity.

Octenol is used as an aggregation pheromone in the sawtoothed grain beetle, *Oryzaephilus surinamensis*^42^ and as a plant attractant in the black blowfly, *Phormia regina*^43^, the legume pod borer, *Maruca vitrata*^44^, the Grapevine Moth, *Lobesia botrana*^45^, the European grape berry moths, *Eupoecilia ambiguella*^46^ and the sandfly *Lutzomyia longipalpis*^47^. It is also used as a compost attractant for the phorid fly, *Megaselia halterata*^48^ and as an avoidance cue for the parasitoid *Lariophagus distinguendus*^49^.

In Diptera, octenol has been suggested to act as an oviposition attractant. First mentioned as a potential oviposition cue for *Ae. aegypti*^50^ and later for *T. amboinensis*^51^, it has also been implicated as an oviposition cue in other dipterans including the oriental fruit fly, *Bactrocera dorsalis*^52^ and the bean seed fly, *Delia platura*^53^. It therefore appears that octenol detection may be an ancestral cue in insects and an oviposition cue in Dipterans^53^.

What is the role of OR8 in *Ae. aegypti*? The ecological context in which AaOR8 operates may be restricted to animal host-seeking, but perhaps more intriguing is the possibility that AaOR8 is involved in eliciting multiple behaviors. As a ubiquitous cue, octenol may be used by mosquitoes in combination with other cues, olfactory or otherwise^54^ to detect a variety of resources important for the life cycle of adult mosquitoes. For example, octenol in combination with CO_2_ and other volatiles may be used as an animal host attractant^5^. But more generally, these insects may use octenol as a proxy chemical cue for detecting humid microhabitats^55^, signaled by the presence of microorganisms such as fungi or bacteria, including oviposition/resting sites and nectar sources (Fig. 1a). Additional studies exploring the behavioral influence of (*R*)-octenol on blood-feeding mosquitoes will be necessary to reveal the entire ecological contexts of its biochemical function.

## Methods

### Gene cloning and sequencing of TaOr8 and TaORco

RNA was isolated from antennae or maxillary palps of adult female *Toxorhynchites amboinensis* by trizol extraction. First strand cDNA synthesis was carried out using the Transcriptor^TM^ kit (Roche Diagnostics, Indianapolis, IN, USA), according to the manufacturers protocol. PCR amplification of full-length *TaOr8* or *TaORco* coding sequences was performed with antennal or maxillary palp-derived cDNA templates and the following primers: *TaORco* forward: 5′CACCATGAATGTTCAACCAACCAAG3′; *TaORco* reverse: TTACTTCAGCTGCACCAGCAC; *TaOr8* forward: 5′CACCATGAGACTCAGAAAGATGAACG3′; *TaOr8* reverse: 5′CTATTTCGGTCCATACATTGTT3′. Amplicons were cloned into the pENTR^TM^ vector using the Gateway^R^ directional cloning system (Invitrogen Corp., Carlsbad, CA, USA) and subcloned into the *Xenopus laevis* expression destination vector, pSP64t RFA.

Plasmids were purified using the The ZR Plasmid Miniprep™-Classic (Zymo Research, Irvine, CA, USA) and sequenced by Macrogen Europe (Amsterdam, the Netherland). DNA and amino-acid sequences for TaOr8 and TaORco have previously been published^27^ and can be accessed here (http://dx.doi.org/10.6084/m9.figshare.1092617).

### Chemical reagents

For establishing the tuning curve, we used the following 29 chemicals, including (*R*)-octenol (described below): 17 compounds from Sigma-Aldrich (Milwaukee, WI, USA), including 1-hepten-3-ol (CAS 4938-52-7), 3-methylbutanol (CAS 123-51-3), *E*-2-hexen-1-al (CAS 6728-26-3), heptaldehyde (CAS 111-71-7), octanal (CAS 124-13-0), propyl-acetate (CAS 109-60-4), 3-octanone (CAS 106-68-3), 6-methyl-5-hepten-2-one (CAS 110-93-0), 2,4,5-trimethylthiazole (CAS 13623-11-5), diallyl-sulfide (CAS 2179-57-9), benzaldehyde (CAS 100-52-7), indole (CAS 83-34-1), histamine (CAS 51-45-6), (+)-limonene oxide (CAS 203719-54-4), geranyl-acetate (CAS 105-87-3), (+)-fenchone (CAS 4695-62-9), 2-oxopentanoic acid (CAS 1821-02-9); 7 compounds from Merck (Darmstadt, Germany), including methyloctanoate (CAS 111-11-5), ethyl-hexanoate (CAS 123-66-0), 2-heptanone (CAS 110-43-0), dimethyl-sulfide (CAS 2179-57-9), tryptamine (CAS 61-54-1), octanoic-acid (CAS 124-07-2) and D-glucuronolactone (CAS 32449-92-6); 2 compounds from Acros Organics (Thermo Fisher Scientific, Waltham, MA, USA), including methyl-salicylate (CAS 119-36-8) and octopamine (CAS 770-05-8); and 2 compounds from Alfa-Aesar (Ward Hill, MA, USA), including L-lactic acid (CAS 79-33-4) and δ-Decalactone (CAS 705-86-2).

Racemic octenol (CAS number 3391-86-4) and *N*,*N*-Diethyl-*m*-toluamide (DEET; CAS number 134-62-3) were obtained from Sigma. (*R*)-(−)-1-octen-3-ol (CAS 3687-48-7, 98.2%) and (*S*)-(+)-1-octen-3-ol (CAS 24587-53-9, >99.9%) chiral compounds were gifts from Bedoukian Research Inc.

### Two-electrode voltage clamp electrophysiological recording of *Xenopus* oocytes expressing TaOR8 and TaORco

The methodologies and protocols used in this study have been described elsewhere^28^. TaOr8 and TaORco cRNA were synthesized using the mMESSAGE mMACHINE^®^ SP6 Transcription Kit (ThermoFisher Scientific) and linearized pSP64tRFA expression vectors. Stage V-VI oocytes were manually separated and enzymatically defolliculated using a 2 mg/mL collagenase (Sigma-Aldrich, Milwaukee, WI, USA) solution (calcium-free ND96 buffer, [pH 7.6]) for 30 min at 18 °C. Oocytes were then successively washed in calcium-free ND96 and gentamycin-supplemented (10 mg/mL, Sigma-Aldrich, Milwaukee, WI, USA) calcium-free ND96. Oocytes were then washed and incubated in ND96 buffer supplemented with calcium (0.1 M), 5% heat-inactivated horse serum (ThermoFisher Scientific), 50 mg/ml tetracycline (Carl Roth GmbH), 100 mg/ml streptomycin (Sigma-Aldrich, Milwaukee, WI, USA) and 550 mg/ml sodium pyruvate (Sigma-Aldrich, Milwaukee, WI, USA) for four to five days. Oocytes were injected with 27.6 nL (27.6 ng of each cRNA) of RNA using the Nanoliter 2010 injector (World Precision Instruments, Inc., Sarasota, FL, USA). Odorant-induced currents of oocytes expressing *TaOr8* and *TaORco* were recorded using the two-microelectrode voltage-clamp technique (TEVC). The OC-725C oocyte clamp (Warner Instruments, LLC, Hamden, CT, USA) maintained a −80 mV holding potential.

For the establishment of concentration-response curves, oocytes were exposed to (*R*), (*S*) or (*RS*)-octenol alone (10^−10^ M to 10^−3^ M). To measure the effect of DEET on TaOR8, we used (10^−7^ M) (*R*)-octenol or a combination of (10^−7^ M) (*R*)-octenol and DEET (10^−3^ M) in 1% DMSO for 8 s. Current was allowed to return to baseline between drug administrations. Data acquisition and analysis were carried out with the Digidata 1550 A digitizer and pCLAMP10 software (Molecular Devices, Sunnyvale, CA, USA).

The tuning curve was generated using a panel 29 odorants including (*R*)-octenol and known to elicit physiological or behavioral responses in mosquitoes (see list of chemicals above). All chemicals used were administered at 400 nM, which corresponds to the EC50 of (*R*)-octenol. All the data analyses were performed using GraphPad Prism 5 (GraphPad Software Inc., La Jolla, CA, USA).

## Additional Information

**How to cite this article**: Dekel, A. *et al.* Evolutionarily conserved odorant receptor function questions ecological context of octenol role in mosquitoes. *Sci. Rep.* **6**, 37330; doi: 10.1038/srep37330 (2016).

**Publisher’s note**: Springer Nature remains neutral with regard to jurisdictional claims in published maps and institutional affiliations.
